# Community-Based Cognitive Social Capital and Self-Rated Health among Older Chinese Adults: The Moderating Effects of Education

**DOI:** 10.3390/ijerph16152741

**Published:** 2019-07-31

**Authors:** Jingyue Zhang, Shicun Xu, Nan Lu

**Affiliations:** 1 Department of Sociology, School of Philosophy and Sociology, Jilin University, Changchun 130012, China; 2 Institute of Gender and Culture, Changchun Normal University, Changchun 130052, China; 3 Department of Population, Resources and Environment, Northeast Asian Studies College, Jilin University, Changchun 130012, China; 4 Department of Social Work and Social Policy, School of Sociology and Population Studies, Renmin University of China, Beijing 100872, China; 5 Sau Po Centre on Ageing, The University of Hong Kong, Hong Kong

**Keywords:** education, social capital, self-rated health, China

## Abstract

This study investigated the moderating role of education on the association between community-based cognitive social capital and self-rated health among older adults in urban Chinese communities. Data were derived from a community survey conducted in Suzhou, China, in November 2015. A sample of 456 respondents aged 60 or older completed interviews. Multiple-group analysis from a structural equation modeling perspective was adopted to examine the proposed model. The measurement model of community-based cognitive social capital featured four trust and reciprocity indicators. Measurement invariance was established across high and low education groups. Education was found to have a moderating effect on the association between community-based cognitive social capital and self-rated health, but only in the high education group. Education should be considered an important factor in future social capital policy and intervention plans. Policy and intervention implications are discussed.

## 1. Introduction

In the past few decades, China has experienced a process of rapid population aging. The population aged 60 or older exceeded 240 million in 2017, accounting for around 17.3% of the total population [1]. China’s rapid aging process is occurring while the local economy is still developing. How can China use its relatively limited social and financial resources to achieve healthy aging in the largest older populations in the world? This is one of the greatest challenges for the Chinese government and society [2].

Self-rated health (SRH) is a comprehensive concept. SRH encompasses biologic, psychologic, and sociodemographic factors and can be used to evaluate individuals’ overall health status [3,4]. Questions concerning SRH not only do not need professional physicians’ assessments, but also have no limitations regarding cultural and social contexts [5,6,7]. SRH was found to be a significant predictor of various health outcomes, including morbidity and mortality [5,8,9]. Therefore, SRH has been extensively used in evaluating older people’s health [5,10].

Various social determinants could affect an individual’s SRH, including sociodemographic characteristics, socioeconomic status, physical health, and living environment [9,11,12,13]. Many of those factors are difficult to modify among older people. The literature has shown that social capital is an important social resource for promoting SRH among older adults and can be modified and changed in later life [14,15]. However, there are three major research gaps in the literature on social capital and SRH. First, there is no unified and consistent criteria for the concept and measurement of social capital. Thus, it is difficult to make meaningful comparisons of findings among social capital studies [15,16]. Many studies ignored the multidimensional nature of social capital and used a single indicator to represent social capital, which might lead to biased findings. Second, the relationship between social capital and SRH might be varied among individuals with different educational backgrounds [17]. Most social capital studies did not consider such potential moderating effects. Third, many social capital studies were conducted in the contexts of Western societies. Because the role of social capital might vary across different social and cultural backgrounds, it is important to conduct relevant studies in East Asian societies and provide local empirical evidence for future policy and intervention designs. Considering these limitations, the present study chose a more comprehensive measurement by building a latent construct of social capital and further examined the moderating role of education in the relationship between social capital and SRH of older adults in urban China.

### 1.1. Defining Social Capital

Social capital is a multifaceted concept. Researchers in different disciplinary domains have different definitions of social capital. This has led to a lack of a unified definition and measurement framework for social capital. In the health field, the most adopted definition was conceptualized by Robert D. Putnam, who described social capital as “features of social organization, such as trust, norms, and networks, that can improve the efficiency of society by facilitating coordinated actions” [18]. This defined social capital from a collectivist perspective. From an individualist perspective, Bourdieu [19] conceptualized social capital as a form of capital based on individuals’ organization memberships, which can be used to understand and explain and individuals’ personal and collective goal achievements. Coleman defined social capital as important social resources in an individual’s social network, where they share social norms and cultural values, common memberships, trust, and reciprocity [20]. While previous scholars put great emphasis on the density or closure of social networks and how they could be used to preserve social resources, Lin [21] examined the crucial role of bridges in searching social resources and exchanging and transferring information from external social networks.

Regarding measurement, social capital is measured in terms of cognitive and structural dimensions. Cognitive social capital refers to individuals’ subjective appraisals, such as trust in the local community and norms of reciprocity with neighbors or friends [22]. Structural dimensions refer to objective measures, including organization membership, civic organizations, and volunteer activity participation [23,24].

### 1.2. Social Capital and SRH

The literature has shown that social capital is an important determinant of older adults’ health, especially individual-level cognitive social capital [2,25]. Studies have found a positive association between cognitive social capital and individuals’ SRH [26,27]. For example, empirical evidence shows that social trust has a contextual effect on individuals’ SRH; individuals who hold relatively lower levels of social trust tend to report lower SRH scores [25]. At the same time, both perceived helpfulness and good social cohesion have a positive effect on individuals’ health outcomes [28]. Similarly, other scholars found that after controlling for risk factors, individuals living in communities with low social capital tended to report poor SRH [29]. Social capital may influence individuals’ health through two pathways. First, social capital could influence individuals’ health-related behaviors through diffusing health-related information; thus, individuals with good social capital could adopt healthy norms and control poor health-related behavior more easily. Second, communities with high social capital likely have better social cohesion to ensure better local services, such as medical treatment, care services, and amenities [30]. In contrast, poor social capital might affect individuals’ feelings of security and lead to low self-esteem, which could have adverse effects on their neuroendocrine states and ultimately influence their physical health [31].

Many studies have proven that cognitive social capital has a positive effect on individuals’ SRH [26,27]. However, findings on the relationship between structural social capital and SRH have been inconclusive. The literature has shown that compared to structural social capital, cognitive social capital has a stronger effect on individuals’ SRH [14,32]. Therefore, in this study, we mainly focused on the relationship between community-based cognitive social capital (hereafter CCSC) and SRH.

### 1.3. Role of Education

Most studies of social capital and SRH only treated educational attainment as a confounding factor [33,34]. Research is lacking on how education moderates the relationship between social capital and SRH [35].

Empirical evidence shows that education levels significantly affect SRH [3,7]. Compared to older adults with lower educational attainment, those with higher educational attainment report better SRH [36]. First, educational attainment could reflect individuals’ health knowledge. Older adults with higher educational attainment likely possess more knowledge related to good health status and can build and maintain healthy behaviors through that health knowledge [35]. Second, older adults with higher educational attainment tend to have higher socioeconomic status. This means that they could have more access to medical resources and enjoy higher nutrition levels [37]. Therefore, SRH is likely to be different among older adults with different education levels.

Furthermore, educational attainment is recognized as an important social determinant of social capital [33,38]. Generally speaking, educational attainment is a proxy for individuals’ relative status—it is a sorting mechanism that could indicate the social capital level of individuals [39]. Individuals with relatively low education levels tend to interact with people close to them; thus, their social networks are relatively small. In contrast, more educated people tend to have wider social networks and higher levels of social trust, reciprocity, and social participation [38,40].

Therefore, social capital could have different effects on individuals’ SRH among various educational attainment groups [35,41]. For more educated people, social capital may help them obtain more health-related information and promote their healthy behaviors [42].

Based on this evidence, we put forward the following hypothesis: Educational attainment moderates the relationship of CCSC and SRH of older Chinese people. Specifically speaking, older adults with relatively high education levels would have more benefits from community social capital.

## 2. Materials and Methods

### 2.1. Sampling

In this research, we conducted a secondary data analysis using community data collected by the Department of Social Work of Renmin University, China. This survey was conducted in the Gusu district of Suzhou, a city in Jiangsu province of China, in November 2015. Ethics approval has been obtained from the Ethics Committee of the University of Hong Kong (Reference No. EA1604030). The aim of the survey was to assess social capital and mental health among community-dwelling older adults. Gusu is in the central area of Suzhou, and 25% of the local residents are aged 60 or older [43].

The survey team used a quota sampling method to collect data. The researchers selected one to two communities from each of the 16 streets in Gusu. In each selected community, they recruited 25 respondents aged 60 or older based on the referrals of community centers and the committee on aging. The age and gender ratio were consistent with those of the local representative sample in Gusu district, which was based on the sixth national census. Other eligibility criteria included having a household registration in Suzhou, living in the community for more than 180 days in the past year, and having adequate cognitive capacities to complete the survey.

The principal investigator provided professional training for around 30 interviewers. The standardized training sessions included the study rationale, questionnaire design, screening questionnaire, obtaining informed consent forms, interview strategies (questionnaire logic, coding of items, patterns of potential answers and potential barriers), quality assurance, data entry, and data cleaning. During the data collection process, principle investigators and supervisors were responsible for checking the accuracy of screening results and recording missingness and errors and other project administration tasks.

The trained interviewers conducted face-to-face interviews with respondents either at their home or in local community centers. A sample of 456 respondents completed the interviews. The survey collected respondents’ demographic characteristics, socioeconomic status, mental health, physical health, and social capital. Specifically, demographic characteristics included age, gender, marital status, and living arrangements. Socioeconomic status was assessed by educational attainments and household monthly income. Mental health was assessed by life satisfaction and depressive symptoms, while physical health included items of chronic diseases and self-rated health. Social capital included items of social trust, reciprocity, belonging to local communities, social participation, organization memberships, and volunteering. In this secondary analysis, we only included data for respondents who completed all questions about CCSC. This generated a final sample size of 439 respondents. 

### 2.2. Measurements

#### 2.2.1. Dependent Variable

The dependent variable of the research was SRH, an individual’s subjective self-evaluation of their overall health [5]. The survey assessed respondents’ SRH through a single question: “How do you think about your overall health status?” Although a simple measure, SRH is an accurate indicator for individuals’ global health and a significant predictor of mortality [5]. SRH is measured on a 4-point scale: 1—poor, 2—fair, 3—good, and 4—excellent. We examined the distribution of SRH and found it was positively skewed. To adjust for skewness, we recoded SRH as a binary variable (0—fair or poor, 1—excellent or good).

#### 2.2.2. Independent Variable

In this study, we used indicators of trust and reciprocity to construct the latent variable of CCSC [23]. Trust was measured by one item: “Generally speaking, the majority of local residents living in this community can be trusted.” Reciprocity was measured by three items: “Local community is a big family and you consider yourself as a member of the big family”; “Local residents help one another out”; and “Local residents care about both their benefits and others’ interests.” We considered these variables to reflect feelings of belongingness, perceived helpfulness of others, and willingness to cooperate with others, respectively. Answers to the four items were measured on a 5-point Likert-type scale. For the items measuring trust, feelings of belongingness, and willingness to cooperate, the scale ranged from 1—strongly disagree, 3—neutral to 5—strongly agree. For the item measuring perceived helpfulness of others, the scale ranged from 1—never helps, 3—sometimes helps to 5—always helps. Higher scores indicate higher levels of CCSC.

#### 2.2.3. Moderator Variable

In this research, education was considered as a moderator variable. Respondents were asked to report their education level. To test the moderator role of education in the relationship between CCSC and SRH, we recoded education level as a binary variable (0—primary school or lower, 1—secondary school or higher). Respondents with secondary school or higher education level were categorized into the high education group. Other respondents were classified in the low education group.

#### 2.2.4. Control Variables

The control variables in the model included basic demographic variables—such as age, gender, marital status, and the living status of older participants (living alone, number of children, and family social capital)—and physical health indicators (activities of daily living (ADLs) and number of chronic diseases). Age was measured in years. Gender and marital status were coded as binary variables (1—female, 0—male; 1—married, 0—other). Financial satisfaction was measured by one item: “In the recent 3 months, did you have adequate money to support your life?” The answers were measured on a 5-point Likert-type scale (1—strongly adequate, 3—fair, 5—strongly inadequate). Living alone was coded as a binary variable (1—yes, 0—no). The respondents were asked to report how many living sons and daughters they had. The Multidimensional Scale of Perceived Social Support [44] was used to measure family social capital (Cronbach’s alpha = 0.917). We used four items to measure family social capital: (a) My family members are willing to help me when necessary; (b) I can discuss important issues with my family members; (c) I can receive emotional support from my family members; and (d) My family members are willing to help me in decision making regarding important issues. The answers were evaluated by a 5-point Likert scale (1—strongly disagree, 3—neutral, 5—strongly agree). We used the mean score of the items to indicate the level of family social capital. Higher scores suggested higher quality of family social capital. The survey listed six of the most common chronic diseases among older Chinese people and required respondents to report whether they had each disease (0—no, 1—yes). We summed the scores, with higher scores indicating worse physical health. Finally, we used the Barthel Index [45] to measure older people’s ADLs, including walking, eating, going to toilet, washing face and brushing teeth, bathing, dressing, getting out of chair or bed, going up and down stairs, and controlling bladder and bowels. Level of independence for each activity was measured by a 10-point scale (0—very difficult, no ability to complete the activity independently; 5—difficult, need assistance; 10—no difficulty). We used summed scores to measure overall ADLs of respondents, with a theoretical range of 0–100 (Cronbach’s alpha = 0.814). Higher scores indicate greater independence in ADLs.

### 2.3. Data Analysis

In this paper, we used multiple-group analysis from a structural equation modeling perspective to analyze the moderator role of education in the relationship between CCSC and SRH. As a moderator variable, education was divided into two groups: low education and high education. We used Mplus 7.0 (Muthén & Muthén, Los Angeles, CA, USA) to conduct the data analysis [46]. Given SRH was treated as a binary variable, the default estimator was diagonally weighted least squares, which is suitable for conducting structural equation modeling with ordinal variables. Research data are available in the Supplementary Materials, File S1.

In the first step, we established the latent variable of CCSC for the two education groups separately by conducting a confirmatory factor analysis [47]. We used the chi-square test statistic, Tucker–Lewis index (TLI), comparative fit index (CFI), root mean square error of approximation (RMSEA), standardized root mean square residual (SRMR), and weighted root mean square residual (WRMR) [47] to evaluate the model fit. The following criteria were used to assess the model fit: non-significant chi-square test estimates (*p* > 0.05), CFI and TLI estimates greater than 0.95, SRMR estimates lower than 0.05, and RMSEA estimates lower than 0.05 suggested good model fit [47,48].

In the second step, to make meaningful comparisons between the two education groups, we tested the measurement invariance across the groups. Measurement invariance can be conceptualized as having four levels: configural, factor loading, intercept, and residual invariance [49]. Factor loading invariance is required to make meaningful comparisons of regression coefficients. We used the chi-square difference statistic and changes in CFI (∆CFI) as criteria to test the measurement invariance [50]. Nonsignificant chi-square values (*p* > 0.05) and ∆CFI larger than −0.01 suggested that measurement invariance was established. Estimates of composite reliability (CR) and average variance extracted (AVE) were also calculated [51]. AVE scores greater than 0.36 (especially when CR value is greater than 0.6) and CR scores greater than 0.7 were acceptable [51]. In the third step, we added SRH and control variables to the model. Wald tests were used to test the moderation effect of education [46]. Finally, we conducted a sensitivity analysis by dividing older respondents into the two education groups (0—secondary school or lower, 1—high school or higher). In this case, the low education group consisted of 156 respondents and the high education group consisted of 283 respondents. They generated similar results.

## 3. Results

### 3.1. Descriptive Statistics

The characteristics of the sample are shown in Table 1. The mean age of the respondents was 70.7 years, 55.6% were female, 76.1% were married, 72% felt they have adequate money to support their lives, and 16.4% were living alone. The mean of number of children was 1.9. The mean family social capital was 4.3. On average, each respondent had 1.21 chronic diseases. The mean score for ADLs was 98.9, meaning that most of the respondents had independence in daily life. Regarding education, 15.9% of the respondents were categorized into the low education group and 84.1% were categorized into the high education group.

Independent *t*-tests and chi-square tests were conducted to test differences in continuous and categorical variables between the two education groups. No significant differences emerged between the two groups in terms of age, self-rated economic status, family social capital, physical health, and ADLs. However, respondents from the low education group tended to have more children (2.46 vs. 1.82) and live alone. Respondents with higher education levels were more likely to be male and married.

### 3.2. Measurement Model of CCSC

In the first step, we separately constructed CCSC measurement models for the two education groups. We used model fit indexes to estimate the model fit, and the values showed that in both education groups, the models fit the data adequately (low: χ^2^(26) = 30.894, *p* = 0.2322, RMSEA = 0.052, CFI = 0.943, TLI = 0.916, SRMR = 0.049; high: χ^2^(27) = 36.058, *p* = 0.1140, RMSEA = 0.030, CFI = 0.979, TLI = 0.967, SRMR = 0.026). In the low education group, the estimates of standardized factor loadings ranged from 0.519–0.753, whereas in the high education group, they ranged from 0.466–0.706.

In the second step, we did not control for any covariates or parameters, and ran the measurement model in both education groups. The model fit indexes were as follows: χ^2^(1) = 1.215, *p* = 0.2704, RMSEA = 0.031, CFI = 1.000, TLI = 0.994, SRMR = 0.008. Then we held the factor loadings equal and reran the model in the two groups: χ^2^(5) = 8.192, *p* = 0.1460, RMSEA = 0.054, CFI = 0.993, TLI = 0.982, SRMR = 0.066. The changed model fit showed that the factor loading invariance was established, because the changed value of CFI was greater than −0.01 and the increased chi-square estimate was not significant. To further test the factor loading invariance, we added the control variables to the model while holding the factor loadings equal in the two groups, and the conclusion did not change. This confirmed that we could compare the regression coefficients of the two groups. Finally, CR and AVE estimates were acceptable in both education groups (low: CR = 0.742, AVE = 0.428; high: CR = 0.738, AVE = 0.428). The main results of the measurement model for CCSC are presented in Table 2.

### 3.3. Moderation Effect of Education

**Figure 1:**
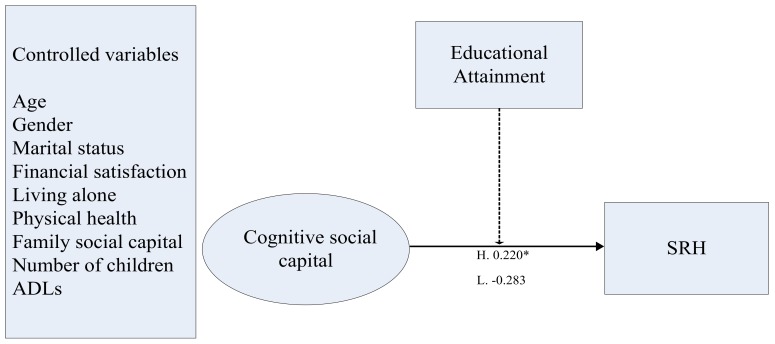
Final model of the role of educational attainment in the relationship between CCSC and self-rated health (SRH). Notes: standardized coefficients are reported. The dashed line indicates a moderating effect; * *p* < 0.05 (two-tailed).

In the final step, we entered SRH and control variables into the model. The estimates of the model fit indexes were as follows: χ^2^(65) = 68.598, *p* = 0.3564, RMSEA = 0.016, CFI = 0.993, TLI = 0.988, WRMR = 0.824. The standardized estimates for the factor loadings ranged from 0.493–0.758. In the high education group, CCSC had a significant influence on SRH (β = 0.626, *SD* = 0.256, *p* < 0.05). In the low education group, the relationship between CCSC and SRH was not statistically significant (β = −0.776, *SD* = 0.464, *p* > 0.05). We conducted a Wald test to compare the regression coefficients of CCSC and SRH, and the resulting estimate showed significant differences between the two groups: χ^2^(1) = 6.819, *p* < 0.01. The structure of the final model is presented in Figure 1.

## 4. Discussion

From the macro perspective, fostering community social capital not only could help older adults achieve the goal of aging in place, but also could be considered as a preventive strategy that could promote healthy aging and sustain older adults’ welfare [1,2]. From the micro perspective, older Chinese adults face enormous challenges caused by social transitions regarding traditional multigenerational family structure, filial culture, and family support. Under such circumstances, fostering community social capital could promote older adults’ health-related behaviors through increasing their rates of social participation and providing better quality of care services, which could further promote their SRH. Results from this study suggest strategies to build suitable community social capital for older adults with different levels of educational attainment and enhance the efficiency of community social capital policy and interventions. This study attempted to examine the moderating role of education in the relationship between CCSC and SRH in the context of urban China. Its findings add new empirical evidence for applying the theory of social capital from an East Asian perspective. The findings also provide evidence and new guidance for policy makers and intervention designers to develop prevention policies focused on building community social capital and enhancing older adults’ SRH in late life.

Consistent with previous findings [25,26,27,29], the findings of the present study show that CCSC had a significant effect on SRH. Although education was found to be significantly associated with SRH [3,7], most previous studies only treated education as a confounding variable [33,34]. This study added new evidence, finding that education had a moderating effect in the association between CCSC and SRH. CCSC was found to have a small effect on SRH among the high education group. No significant effect of CCSC on SRH was found among the low education group. Therefore, the conclusion of this study supported the hypothesis. Older adults with relatively high education levels might accumulate more social, medical, and financial resources compared to their less-educated counterparts. These individuals are in a better position to use these resources, which might lead to better nutrition and fewer diseases. This has accumulative effects on population health. Furthermore, older adults with higher educational attainment could have better capacities to utilize health services, gather health-related information, and learn new knowledge and skills than those with low educational attainment [38,39,40]. Therefore, educational attainment among older people should be considered in the development of community social capital interventions. Different social organizations and activities should be designed to remove potential barriers and meet the social needs among older adults with different education levels. Specifically, the findings of this study show that community social capital is more suitable for older adults with higher educational attainment. In this case, for older people in the low education group, we should consider how to build individualized community social capital services to improve their overall health status.

Based on the findings of the present study, we propose the following policy and intervention implications. First, community social capital should be considered as a screening instrument to identity older people at risk of poor SRH in urban communities. Community-dwelling older people with lower education levels are also at risk of poor SRH. Therefore, these older adults should receive attention in the development of social capital policies and interventions. For example, community organizations should develop peer-support programs to encourage older adults with relatively high education levels to share knowledge and train other older residents in terms of health-related information and skills. This could not only promote self-efficacy among older adults, but also improve the efficiency of social capital interventions. Second, the findings of this study support the idea of lifelong learning among older populations. Communities could conduct relevant educational programs around health-related and nutrition knowledge among older adults through various organizational formats. Especially for older adults with lower education levels, besides diffusing health-related and nutrition knowledge, these community-based programs could enhance their basic survival skills. Qualitative studies are needed to examine the potential barriers that these older adults might encounter when they utilize community social capital to enhance their health. Furthermore, the interplay between cognitive social capital and structural social capital and their influences on SRH should be further examined in future longitudinal studies with larger sample sizes.

This study had some limitations. First, the nature of cross-sectional data did not allow us to test the direct causal relationship among CCSC, educational attainment, and SRH. Older adults with better SRH are more likely to have higher social participation and foster higher social trust with others from local communities. Longitudinal studies with larger samples are needed to further examine these causal relationships and the moderating role of education. Second, random sampling methods were not been applied in the recruitment of the respondents. Therefore, the empirical generalization of the findings should be limited to populations with similar social and cultural backgrounds. Future studies should be conducted to test the measurement model of CCSC in other small cities and rural areas. Finally, the low education group was relatively small and predominantly female. This limits the generalization of the findings. Future studies with lager sample sizes are needed to examine how social capital influences the welfare of older adults with low educational attainment.

## 5. Conclusions

This study aimed to examine the moderating role of education level on the relationship between CCSC and SRH among Chinese urban older adults. We built the latent variable of CCSC and conducted a multiple-group analysis. The results showed that CCSC was a significant indicator of individuals’ SRH in the high education group; however, CCSC had no significant effect on older people in the low education group. Policy makers and intervention designers should consider using social capital instruments to identity high-risk populations, especially older adults with low educational attainment. CCSC is an important indicator of individuals’ SRH. Potential barriers that might prevent older adults with low educational attainment from having access to social supportive resources in local communities should be removed. Some projects could be developed to enhance trust and reciprocal exchanges of older people, including intergenerational mutual assistance programs, provision of financial incentives, and peer-support groups in local communities.

## Figures and Tables

**Table 1:** Sample characteristics.

Characteristics	Full Sample (*n* = 439)		High EL Group (*n* = 369)		Low EL Group (*n* = 70)	
	*n* (%)	Mean (SD)	*n* (%)	Mean (SD)	*n* (%)	Mean (SD)
Age		70.7 (7.3)		70.0 (7.0)		74.64 (7.6)
Gender						
Male	195 (44.4)		179 (48.5)		16 (22.9)	
Female	244 (55.6)		190 (51.5)		54 (77.1)	
Marital status						
Married	334 (76.1)		294 (79.7)		40 (57.1)	
Other marital status	105 (23.9)		75 (20.3)		30 (42.9)	
Financial satisfaction						
Adequate	316 (72.0)		272 (73.7)		44 (62.9)	
Fair	85 (19.4)		70 (19.0)		15 (21.4)	
Inadequate	38 (8.5)		27 (7.3)		11 (15.7)	
Living alone						
Yes	72 (16.4)		51 (13.8)		21 (30.0)	
No	367 (83.6)		318 (86.2)		49 (70.0)	
Physical health	1.2 (1.0)			1.2 (1.1)		1.1 (0.9)
Family social capital	4.3 (0.8)			4.3 (0.7)		4.1 (0.8)
Number of children	1.9 (1.1)			1.8 (1.0)		2.5 (1.2)
ADLs	98.9 (4.7)			98.9 (4.8)		98.5 (4.5)

Note: EL—education level; SD—standard deviation; ADLs—activities of daily living; ADLs were assessed by the Barthel Index; variable ranges: age (60–92); gender (1–2); marital status (0–1); self-rated economic status (1–5); living alone (0–1); physical health (0–6); family social capital (1–5); number of children (0–11); ADLs (0–100).

**Table 2:** Measurement model of CCSC.

Factor Indicator	Estimate	SD	Standardized Estimate	SD
CCSC (Low education level group)				
Trust in local community	1.000	0.000	0.477 ***	0.076
Perceived helpfulness of others	1.589 ***	0.216	0.636 ***	0.073
Willingness to cooperate with others	1.825 ***	0.246	0.625 ***	0.063
Feelings of belonging	1.696 ***	0.251	0.830 ***	0.065
CCSC (High education level group)				
Trust in local community	1.000	0.000	0.413 ***	0.047
Perceived helpfulness of others	1.589 ***	0.216	0.578 ***	0.049
Willingness to cooperate with others	1.825 ***	0.246	0.690 ***	0.047
Feelings of belonging	1.696 ***	0.251	0.856 ***	0.052

Notes: *** *p* < 0.001 (two-tailed); CCSC—community-based cognitive social capital; SD—standard deviation.

## Supplementary Materials

The following are available online at https://www.mdpi.com/1660-4601/16/15/2741/s1. File S1: Research Data.
